# Indents

**Published:** 1915-09

**Authors:** 


### INDENTS

☞ **Brief Articles of Technical or Scientific Value will receive notice and comment. Contributions invited.**

*Preliminary Treatment of Peridentitis.* A general irrigation or spraying of the entire oral cavity with a warm disinfectant solution should immediately precede all operative treatment. This for the purpose of cleansing the mouth of superfluous infective material and to render effective the topical application of a stronger disinfectant lotion, such as iodine-phenol compound. Then the instrumentation for the removal of all deposits on the exposed tooth surfaces and the smoothing of rough enamel, etc., should prepare for the polishing of these surfaces which should be done at once if practicable. The patient should then be dismissed for a few days with instructions as to keeping the exposed root and crown surfaces as clean as possible by a proper mouth toilet. If there is much suppuration the emetin treatment, combined with local applications by injection into the pockets of suitable disinfecting solutions, will prepare the case for subsequent root surfacing and alveolar currettement. This should be done as a surgical operation. That is under as complete aseptic conditions as may be practicable, only a few teeth should be thoroughly done at a single sitting, and the wounds should be permitted to heal without further surgical interference if possible. In some cases anesthetics are necessary for thorough operations. The conductive method probably will serve the best purpose. There are advantages in making the operations when practicable without anesthetics. If the anesthetic is necessary to complete the operation it is always indicated.

*Care of French Soldiers Wounded in the Jaws.* The French army surgeons have made special provision for oral surgical care of all jaw cases by recruiting dental men to act on the field in caring for wounds temporarily until the victims can be sent to the hospitals where more permanent treatment can be given. These field dental surgeons will be equipped with supplies and facilities which will enable them to take care of such cases to the best advantage. It is expected that a sufficient number of diploma qualified men can be found in the recruited soldiers to properly put this plan into effective execution.

*To Remove Plaster from Vulcanite Plates.* Place the well-washed plate in a saturated solution of sodium thiosulphate over night. The plate can then be easily washed clean, if it is not already clean when taken from the solution.—*Record.*

*Excess in Carbohydrate Foods.* Excesses in carbohydrates are produced by children eating excessively of jams, sweets, teas, etc. Children should be induced to eat more milk and fats, more nuts and fruits. Excesses in carbohydrate leads to acidosis because of insufficient oxidation of the carbohydrates. Dental caries and dyspepsia are important symptoms of excesses in this direction.—E. J. Tyrell, in *Dental Record.*

*Hot Drinks a Cause of Gingivitis.* R. Ackerly, in *Dental Record,* reports a case where an Arab, adopting the English custom of taking large cups of very hot tea and coffee, developed a very serious case of gingivitis. These cases are relieved readily by stopping the hot drinks.

*Ameba in Pyorrhea.* I have just made a series of experiments, both as to the ameba in pyorrhea pockets, and to the use of emetin hydrochlorid hypodermically in the treatment of pyorrhea. I was skeptical about the ameba being an etiological factor and also that emetin was a specific; but since Barrett and Smith and Bass and Johns were men of reputation, I felt that their theories were worthy of consideration, and that it was unfair not to give the subject due consideration.

In my first series I examined the pus from twenty well-developed pyorrhea cases. In eighteen of these the ameba was present. In one of the cases in which the ameba was not found the patient had just finished a course of potassium iodid and mercury, and to this I attributed the absence of the organism. In the other I was unable to account for the absence of the protozoa. In these eighteen cases I injected hypodermically emetin hydrochlorid, one-half a grain daily for four days in succession, with no other treatment of any kind.

In a few days after the last injection, microscopic examination was made and sixteen of these cases gave negative results. In two the ameba were still present. In some of these cases there was improvement, in others marked improvement.

I then made another series of examinations in *non*-pyorrhea cases. There were twelve in this series, seven of which showed ameba, and five were negative. In four of the negative cases the gums were perfectly healthy and mouth and teeth clean. One had taken alcresta ipecac a few days before. In the non-pyorrhea cases in which ameba were found the gums were red at their margins, but there were no well defined pus pockets.

In four to five weeks after the use of the emetin treatment, in the pyorrhea cases microscopical examinations were again instituted. I then found the ameba just as numerous as before, and what gain there had been in the mouth condition had been lost.—Dr. Thos. L. Gilmer, in *Dental Review.*

*Pyorrhea Alveolaris Begins in the Gums.* Perhaps no more conclusive argument can be adduced on this point than the fact that every dentist has noticed that the removal of infected teeth results ninety-nine times out of a hundred in cessation of all inflammatory symptoms. The second corroborative fact is that removal of the infected porous root surface usually checks all types of interstitial gingivitis quickly if the work be accurately done, but if the tooth's surface be neglected and dirty, gingivitus recurs.

In the observation of more than two thousand cases, the author has found no variation from this general rule, except in cases of acute diffuse nephritis, diabetes-mellitus, and certain types of drug poisoning.—Dr. T. B. Hartzell.

*Cause of Gingivitis.* Dr. Hartzell, in the *Dental Review,* makes the following statement. I believe the bacterial masses on the tooth surface furnish the chief irritant and the imperfectly protected gum margin permits the irritant to enter the tissues, the blood supply being received from end capillaries, the return flow is blocked by swelling, thus creating a favorable field for bacterial invasion. We find the lingual surfaces of the lower central incisors are particularly subject to the accumulation of salivary calculus and it has been the experience of every observing dentist that we have recession of bone and gum on the lingual surfaces of these teeth, accompanied by a comparatively slight inflammatory disturbance with no microscopic evidence of pus, just a low grade gingivitis. It is also true that gingival inflammation does not occur on the gum margins of patients who have consistently practiced oral prophylaxis on the tooth surfaces, so that the tooth surfaces are free from bacterial and mechanical irritants.

*An Unusual Case Treated Unsuccessfully with Emetin.* Dr. Hartzell reports a case of a child of twelve years who had an exaggerated case of pyorrhea who had lost all the upper teeth except the right cuspid and all the lower teeth except the bicuspids and second molars. Twenty injections into the pockets about these teeth and subsequent hypodermic injections of emetin has had no appreciable effect in checking the pus discharge. It is rarely that we have pyorrhea in cases so young. There must be other affections.

*Is the Entamoeba Always Present?* Drs. Hartzell and Henrici, of the University of Minnesota, after careful research, say: "We have not found the entamoeba to be universally present. It is true that we have found the entamoeba in three-fourths of the cases examined, whereas we find the fusiform bacillus and the streptococcus viridans absolutely universal in their presence. In fact, in our search for bacteria-free root ends in living teeth, we discovered that it was necessary to actually burn with a cautery the tissues under the gingival margin if we expected to find the root ends free from bacterial growth, and until we did so begin to sterilize by actually burning the tissues beneath the gum margin, we always, without exception, found the tooth's root infected. Personally, the author believes it to be almost an impossibility to extract a healthy living tooth and find the root free from viridans unless the tooth has been rendered free from bacteria by rubbing the tooth with iodin and by subsequently burning the gingival field with the actual cautery. Even with the above precautions we found living teeth whose roots were infected, showing that the vessels of the peridental membrane form the path through which root ends are most commonly infected."—*Dental Review.*

*Sealing Pyorrhea Pockets After Operating.* Dr. Hartzell, in the September *Review,* gives the following description of Adair's treatment: Take Merck's beechwood creosote saturated with all the iodin it will dissolve. This makes a heavy black oily mixture and is to be applied to the gum edge and to the neck from one-eighth to a quarter of an inch to the gum edge and reaching up on the tooth neck to the enamel of the crown. Carefully preventing the saliva from touching this after it has been placed, we immediately apply a second coat composed of glycerin in which we have incorporated all the tannic acid that it can be made to dissolve. Thus the tannic acid most completely covers the first coat of creosote-iodin, and, if it is placed before moisture comes in contact with the creosote-iodin, the two solutions seem to unite and form a dense, tough, black-brown material which will cling to the tissues from twenty-four to forty-eight hours, protecting the gingival margin from a bacterial incursion for that period of time. If thought advisable by the operator, the coating may be repeated every other day for several days, thus maintaining a condition of approximate asepsis. The author might state in passing that the application of this double coating to sutured incisions in the mouth protects these edges from infection and renders a wound much more comfortable than otherwise would be the case. It may also be applied to the cut edges where third molars have been uncovered by removing the gum. As soon as the bleeding has been checked by the application of hot packs, the cut surfaces may be coated with this heavy iodin solution followed by the tannic acid glycerin solution and the cu surface will be protected from infection and also from the irritation of the movement of the tongue.''

*The Endamoeba Buccalis in Pyorrhea Alveolaris.*

The drug preparations used are (1) hypodermic tablets emetin hydrochloride ½ gr., (2) 1 c.c. ampules containing a solution of ½ gr. emetin hydrochloride in water, (3) 2 c.c. ampules of ½ per cent. solution emetin hydrochloride, (4) alcresta ipecac tablets.

The hypodermic tablets may be administered hypodermically in the arm—dose one tablet per day. Or a ½ per cent. solution for local use may be made by dissolving one tablet in 100 minims of water.

The 1 c.c. ampule containing ½ gr. emetin hydrochloride is intended for hypodermic administration—dose one ampule per day.

The 2 c.c. ampule of ½ per cent. solution of emetin hydrochloride in water is prepared for local use in pyorrhea pockets.

The alcresta ipecac tablets may be given by mouth, without causing nausea—dose 10 gr. tablets, two tablets one-half hour before meals.

Treatment, whether local or general, or both, may be carried on three days continuously, followed by two days' rest, then resumed for three days again.

The danger from hypodermic administration is soreness at the point of administration.

The dangers from the use of the ½ per cent. solution in the mouth are: (1) Nausea from swallowing some of the solution, and (2) stomatitis, of a characteristic nature. Discontinuance of the use of the drug and an oily, antiseptic spray for the mouth will cure the stomatitis promptly.

The danger from the use of alcresta ipecac tablets is that diarrhea may be produced. This is much more likely to occur in men than women. Treat simply by reducing the dose or discontinuing the use of the drug according to the severity of the diarrhea.—Howard R. Raper, in *Dental Review.*

*Forsyth Infirmary.* The editor of Oral Hygiene recently visited Boston and makes this comment on the work of this Institution: "At the time of my visit, late in the month of May, the Infirmary had been open for four months and was running about two-thirds of its capacity. As the organization becomes perfected the full number of chairs will be in operation. The average number of children treated has been approximately three hundred per day; eight thousand different children and three thousand dismissed as completed, with an average number of operations per child, including cleaning, extracting and fillings of 6.4."

*The Amoeba and Pyorrhea.* The medical and dental professions all over the world have been led to believe that the amoeba is the responsible factor for all pyorrheal processes and that its destruction would be a panacea in the cure of pyorrhea, and the author earnestly hopes that this may be true. Nevertheless, his own experience does not lend color to this belief and we are thrown back upon the best teacher we have had in this matter up to the present time, namely, experience, which indicates in no uncertain terms that the gingival margin is the point where pyorrheal inflammations begin and that the pyorrheal inflammations are wrought by and maintained by the poison *proteins induced* by the presence and destruction of mixed bacterial infections by the body ferments, and experience has also taught us that clean tooth surfaces preclude gingival infection and that heavy massage of the gums also tends to harden and render more resistant the gingival margins to infection.—T. B. Hartzell.

*Prophylaxis Treatment of Tooth Surfaces.* Experience has taught us that the logical place to begin the treatment of pyorrhea is the tooth's surface and that the first step in the treatment of any pyorrhea is to demonstrate to the patient and to the operator the relative amount of bacterial growth on the tooth's surface. This is important to them both, important to the patient because it directs his attention to those areas of the tooth's surface which need the greatest daily care, and important to the operator because it explains to him the reason for gingival inflammation where oftentimes no other rational cause can be found. Following such demonstration, the next step should be the elimination of all rough areas on the tooth's surface which may retain bacteria on or in the tooth surface, which is accomplished by applying to them finishing stones, swinging them across the enamel surfaces, thus bringing out the pits that have been induced by the acid formed by the bacteria on these surfaces, getting rid of the bacteria themselves and polishing out the inequalities which serve to retain and foster bacterial growth. After the use of the coarse stones on the tooth surfaces, disclosing stain should be again used to make sure that all pits have been beveled out. After all pits that contain bacteria and inequalities have been beveled out, and the application of the disclosing stain reveals nothing, then the tooth surfaces should be thoroughly polished with the Arkansas stones, and the brilliance increased by the use of moosehide wheels loaded with jeweler's rouge or with any other abrasive which will give a high degree of brilliance to the enamel.—Hartzell, in *Dental Review.*

*Partial Removable Dentures.* Partial removable dentures consist of dentures constructed of the various materials adapted to the special needs of a case. These dentures, whether swaged, cast, or molded, are closely adapted to the form of the model or cast, with the same consideration regarding the relief of the hard areas as is necessary in full dentures. Except in rare cases, plates are assisted in their retention by one of the various gold clasps or special attachments. I emphasize the term "assisted," because the clasps should be used only for that purpose. The ridge and palate portion should be the burden-bearers, with the teeth acting as adjuncts. Not that there should be an overburdening of the ridges, for this has its bad aspect and should be obviated by the construction of a lug or spur upon the occlusal portion of the clasp, according to the plan of Dr. Bonwill.—*Cosmos.*

*Pyorrhea Pockets.* Nearly every writer on the subject of "so-called pyorrhea" has mentioned pus or pyorrheal pockets. Many, in speaking of treatment, scrape away the "dead bone until healthy tissues are reached." I have seen this scraping process continued in the mouth of a young woman of twenty-two years until the apical ends of the six anterior lower teeth were exposed; they then had to be extracted. The sufferings of this young woman while undergoing this operation were terrible. How do these writers know that they are treating "pus pockets"? How do they know that there is "dead bone"?

### Treatment of Pockets.

In the treatment of pockets, the less instrumentation used to remove the deposits upon the roots of the teeth the better. There being no dead bone present, the alveolar margins should not be scraped, since they will take care of themselves. All that is necessary is to give nature a chance. Naturally the alveolar process, owing to its transitory nature, is better prepared to take care of itself than any other bone structure in the body. Instrumentation should be limited to avoid infecting the tissues, should pus germs be present. Much pain inflicted by so-called specialists can thus be avoided.

Halisteresis and perforating canal absorption, producing pockets, may occur around any one of the teeth in the mouths of people over thirty years of age. Pus is only present in a very small percentage of patients; since, therefore, there are many pockets without pus, there should be a new classification of the pathology in connection with this disease.—E. S. Talbot, in *Cosmos.*

*What Next in the Treatment of Pyorrhea Alveolaris?*

So many "sure cures" for this distressing malady have been exploded in the past two or three years that the public are becoming a bit gun-shy. Aside from the fact that a little experience has been gained by my failure, a few rays of light have been shed by these explosions.

The first grenade I know of was Dr. Head's *tartar solvent,* which after many diligent attempts was soon discarded.

The *vaccine treatment* came fast on the heels of the tartar solvent, tarried awhile, then traveled the path of its predecessor. I went into this treatment very assiduously, and concluded after many trials that the small percentage of cures and the difficulty of administration blighted the sanguine hope I held for this remedy.

The vaccine treatment for pyorrhea, like Friedmann's cure for tuberculosis, was heralded as a positive remedy, but when brought before the great judge, Time, it was pronounced worthless for lack of evidence, notwithstanding the fact that Dr. Medalia, of Boston, who experimented much in this field, prefers this method to emetin.

Today we have reached the stage of *emetin hydrochlorid* treatment, and the good that has been obtained so far is remarkable and encouraging. But let us not be too enthusiastic, until Time has passed judgment, otherwise the public will accept nothing in the future that the dentist may suggest to relieve pyorrhea.

One of the most difficult problems that confront the dentist in this treatment is the ability to differentiate between how much the emetin accomplishes and how much instrumentation accomplishes independently of each other. I was told by a reputable dentist that Dr. Bass, of Tulane University, had told him that emetin without instrumentation would effect a cure. This does not seem possible. I heard Dr. Bass speak on this subject, and the one feature that struck me forcibly was his utter disregard for instrumentation; he made no reference to it whatever, although he did make some little mention of it in his article published in the *Journal of the American Medical Association.*

A dentist in my city expressed the opinion that all or nearly all of the improvement noted in his cases was due to instrumentation.

A decided improvement over the injection of emetin hydrochloride is the treatment with alcresta ipecac tablets by the mouth, which yields the same effect.

Dr. Eugene S. Talbot says in his work on ''Interstitial Gingivitis'' that Dr. C. E. Sayre and Dr. A. E. Flower, two practitioners of comparative medicine, when consulted as to the frequency of this disease in animals, said that eighty per cent. of dogs over eight years had pyorrhea. Dr. Bass said in one of his lectures that dogs were not subject to this disease.

In April 10th issue of the *Journal of the American Medical Association* appeared an article by Drs. Wright and White of the navy, giving twenty-eight cases of pyorrhea that were treated with injections of *mercuric succinimid,* and all of which were completely cured in a remarkably short period of time. Will this drug supplant emetin?—Dr. F. P. Kehoe, in *Cosmos.*

*Indictment of the Dentist.* The dental surgeon stands indicted by the general public as the most cruel, barbarous and inhuman of medical specialists. The opthalmologist, otologist, rhinologist, laryngologist, gynaecologist, oral surgeon and other specialists have adopted methods of pain alleviation, thus doing better work and getting better results than formerly, but the rank and file of the dental profession are no more advanced than they were a quarter of a century ago, in the matter of making dental operations less painful.

"It seems almost beyond comprehension, when the means are at hand to prevent pain, and the public are clamoring for it, demanding it, we go on in the same old way, obtaining inferior results, knowing at the time that the operation falls short of what it should be, and will fail in a few months because we can not remove the carious dentine thoroughly or can not prepare a cavity sufficiently well for the retention of a filling or inlay, and fail utterly in shaping a tooth for a crown, and are defeating in removing calcarious deposits, yet 90 per cent. of dental surgeons continue this line of practice."—Dr. Hewitt.

*Ideation Under Anesthesia.* Sir Frederick W. Hewitt's Table, showing the degrees or stages in the action of the chief general anesthetics upon the human organism, and the phenomena that usually characterize these stages, makes twelve subdivisions for the stage of analgesia. Among them is "excessive ideation," "disturbance of judgment" and "dreams." At the time the patient is experiencing "excessive ideation" and "dreams," HELPFUL SUGGESTIONS ARE IN ORDER. "There is a period under an anesthetic at which many patients, indeed most patients, will see, feel and experience the very things suggested as surely as if they were real, and this period is just before consciousness is lost; just at that stage where painless cavity preparation can be made.''
—Dr. Ford.

*The Dentist.* ''He well deserves a laurel wreath, the man who tinkers with my teeth, when they are out of plumb; he plugs them up with melted lead, and soothes my swelled and aching head, and heals the tortured gum. Upon his skill your comfort hangs when you have trouble with your fangs, and seldom does he fail; his shining instrument he wags and draws the old insurgent snags, then draws his slice of kale. No more you hear in dentist's room the shrieks of those who dread their doom, of those whose souls are sick; the patient calmly sits and smiles, the while the dentist with his files and pincers does the trick. How different in olden days! The dentists then had painful ways; he sat upon a bench, and took your head between his knees, and, muttering, 'Look pleasant, please,' he plied his monkey wrench. It took six men to hold me down when he adjusted bridge or crown, or plugged a hollow fang, and travelers could hear me roar away upon the distant shore of Yang-tsze-kiang. But now I like the dentist's chair, I like to sit and rest me there, from morning until dusk, to sit in comfort and to snooze, and have the gentle dentist use his forceps on my tusk.''
—Walt Mason.

